# Supplementary material for: High-fat diet ablates an insulin-responsive pool of GLUT4 glucose transporters in skeletal muscle
Source: bioRxiv. 2025 Jul 3:2025.06.29.662135. Preprint. [Version 1] doi: 10.1101/2025.06.29.662135 (PMC12236751; doi:10.1101/2025.06.29.662135)

bioRxiv preprint doi: https://doi.org/10.1101/2025.06.29.662135; this version posted July 3, 2025. The copyright holder for this preprint (which was not certified by peer review) is the author/funder, who has granted bioRxiv a license to display the preprint in perpetuity. It is made available under aCC-BY 4.0 International license.

## Supplementary Figure Legends

# High fat diet ablates an insulin responsive pool of GLUT4 glucose transporters in skeletal muscle

Youjia Hu[1], Stacey N. Brown[1], Ali Nasiri[1], Don T. Li[1,8], Haiyan Wang[2], Gregory D. Cartee[2,3,4], Gerald I. Shulman[1,5], and Jonathan S. Bogan[1,6,7]

[1]Section of Endocrinology and Metabolism, Department of Internal Medicine, Yale School of Medicine, New Haven, CT 06520-8020, USA
[2]Muscle Biology Laboratory, School of Kinesiology, University of Michigan, Ann Arbor, MI 48109, USA
[3]Department of Molecular and Integrative Physiology, University of Michigan, Ann Arbor, Michigan, USA
[4]Institute of Gerontology, University of Michigan, Ann Arbor, Michigan, USA
[5]Department of Cellular and Molecular Physiology, Yale School of Medicine, New Haven, CT 06520, USA
[6]Department of Cell Biology, Yale School of Medicine, New Haven, CT 06520, USA.
[7]Yale Center for Molecular and Systems Metabolism, Yale School of Medicine, New Haven, CT 06520, USA
[8]Present address: Department of Pediatric Orthopedics, Hospital for Special Surgery, New York, NY 10021, USA

bioRxiv preprint doi: https://doi.org/10.1101/2025.06.29.662135; this version posted July 3, 2025. The copyright holder for this preprint (which was not certified by peer review) is the author/funder, who has granted bioRxiv a license to display the preprint in perpetuity. It is made available under aCC-BY 4.0 International license.

**Suppl. Fig. 1. Supporting data for studies of glucose turnover in MUKO mice.**
**a.** Quadriceps muscles were isolated from WT mice, homogenized, and T-tubule enriched membrane fractions were prepared. Immunoblots were performed as indicated to demonstrate the purity of the fractions.
**b.** GLUT4 was immunoblotted in total homogenates of WT and MUKO mice (Fig. 1c), data were quantified and are plotted. n = 18 mice in each group.
**c.,d.** IRAP and GLUT4 abundances were quantified in T-tubule fractions of WT and MUKO mice (Fig. 1c-e), and the ratio of IRAP to GLUT4 abundance is plotted. Data are plotted for fasted and insulin-stimulated mice (c) and in all WT and MUKO mice (d). In (c), n = 3 mice in each group. In (d), n = 6 mice in each group. NS, not significant.
**e.** Plasma glucose concentrations were measured in 12-week-old WT and MUKO mice fasted for 6 h. n = 11 mice in each group. NS, not significant.
**f.-h.** Body weight (f), fat mass (g), and percent fat mass (j) were measured in 12-week-old mice used for hyperinsulinemic-euglycemic clamps. n = 17 WT and 10 MUKO mice. NS, not significant.
**i.** HOMA-IR was calculated from fasting glucose and insulin concentrations measured prior to hyperinsulinemic-euglycemic clamps. n = 17 WT and 10 MUKO mice.
**j.,k.** Glucose infusion rate (GINF) and plasma glucose concentrations are plotted versus time during the hyperinsulinemic-euglycemic clamp. Mean ±s.e.m., n = 17 WT and 10 MUKO mice.
**l.-s.** The indicated parameters were measured in hyperinsulinemic-euglycemic clamps. n = 17 WT and 10 MUKO mice. EGP, endogenous glucose production. NEFA, nonesterified fatty acid. NS, not significant.
All data are presented as mean ± s.e.m. of biologically independent samples, analyzed using a two-tailed t test (c-h,k-r) or ANOVA with adjustment for multiple comparisons (b).

**Suppl. Fig. 2. Supporting data for studies of energy expenditure in MUKO mice**
**a.** Rate of oxygen consumption ($VO_2$) was measured by in metabolic cages in 13-week-old mice and is plotted over time. n = 21 WT and 17 MUKO mice. Mean ± s.e.m. is shown. Individual time points were analyzed using two-tailed t tests. *$P$<0.05, **$P$<0.01.
**b.** Energy expenditure per mouse is plotted as a linear regression versus total body weight. n = 21 WT and 17 MUKO mice. Data were analyzed by ANCOVA. NS, not significant.
**c.-j.** The indicated parameters were measured in metabolic cages (c-h) or in body composition analyses done immediately before metabolic cage analyses. n = 21 WT and 17 MUKO mice. Data are presented as mean ± s.e.m. of biologically independent samples, analyzed using a two-tailed t test. NS, not significant.

**Suppl. Fig. 3. Supporting data for studies of glucose turnover in HFD-fed MUKO mice.**
**a.-e.** Body weight and composition were measured prior to hyperinsulinemic-euglycemic clamp studies, and the indicated parameters are plotted. n = 13 WT and 14 MUKO mice. Mean ± s.e.m. is shown, analyzed using two-tailed t tests. NS, not significant.
**f.,g.** Glucose infusion rate (GINF) and plasma glucose concentrations are plotted versus time during the hyperinsulinemic-euglycemic clamp. Mean ±s.e.m., n = 13 WT and 14 MUKO mice.
**h.-o.** The indicated parameters were measured in hyperinsulinemic-euglycemic clamps. n = 13 WT and 14 MUKO mice. Data are presented as mean ± s.e.m. of biologically independent samples, analyzed using a two-tailed t test. EGP, endogenous glucose production. NEFA, nonesterified fatty acid. NS, not significant.

**Suppl. Fig. 4. Supporting data for studies of energy expenditure in HFD-fed MUKO mice.**
**a.-i.** Mice were treated with a HFD for 3 weeks, then studied at age 12 weeks using indirect calorimetry and analysis of body weight and composition. The indicated parameters were measured and are plotted. n = 7 WT and 9 MUKO mice.

bioRxiv preprint doi: https://doi.org/10.1101/2025.06.29.662135; this version posted July 3, 2025. The copyright holder for this preprint (which was not certified by peer review) is the author/funder, who has granted bioRxiv a license to display the preprint in perpetuity. It is made available under aCC-BY 4.0 International license.

**j,k.** Mice were fed a HFD beginning at 8 weeks of age. Body weight (j) and GWAT weight (k) were measured after 7 weeks. n = 7 mice in each group.
All data are shown as mean ± s.e.m., analyzed using a two-tailed t test. NS, not significant.

**Suppl. Fig. 5. Supporting data to characterize GLUT4 Storage Vesicle -regulating proteins**
**a.** TUG and flag-tagged AS160 were expressed by transient transfection of HEK293 cells. Cells were lysed and flag-AS160 was immunoprecipitated. Eluates and lysates were immunoblotted as indicated. IP, immunoprecipitate. WB, western blot.
**b.** TUG and flag-tagged TNKS2 proteins were expressed by transient transfection of HEK293 cells. The flag-TNKS2-ANK construct contains only the ankyrin repeat domain of TNKS2. Cells were lysed and flag-TNKS2 proteins were immunoprecipitated. Eluates and lysates were immunoblotted as indicated. IP, immunoprecipitate. WB, western blot.
**c.** Lysates from epitrochlearis muscles of WT and AS160 KO rats were immunoblotted for TUG. Data were quantified and normalized to that in WT controls. n = 7 WT and 8 KO samples.
**d.,e.** T-tubule membrane fractions were isolated from quadriceps muscles of 14-week-old mice that had been maintained on regular chow (RC), or fed a high-fat diet (HFD) for 6 weeks, as described for Fig. 4h-k. As similar numbers of saline- and insulin-treated samples were analyzed in each group, the overall abundance of IRAP (d) and ratio of IRAP to GLUT4 (e) were quantified by genotype. For (d), n = 17 RC and 13 HFD samples. For (e), n = 14 RC and 10 HFD samples.
**f.,g.** T-tubule membrane fractions were previously isolated from saline- and insulin- treated WT and muscle TUG knockout (MTKO) mice, and were immunoblotted to detect IRAP and GLUT4[16]. These previous data were analyzed to quantify the ratio of IRAP to GLUT4 (f). n = 4 saline WT, 3 insulin WT, 4 saline MTKO, and 3 insulin MTKO samples. In (g), data were grouped by genotype.
All data are presented as mean ± s.e.m. of biologically independent samples, analyzed using a two-tailed t test (c,d,e,g) or ANOVA (f). NS, not significant.

**Suppl. Fig. 6. Supporting data for studies of glucose turnover during fasting in high-fat diet -fed muscle-specific TUG knockout mice.**
**a.** HOMA-IR was calculated from 6-h fasted glucose and insulin concentrations prior to glucose turnover studies presented in Fig. 5. n = 13 WT and 7 MTKO mice.
**b.-f.** Body weight and composition were measured and the indicated parameters are plotted. n = 13 WT and 7 MTKO mice.
**g.** Glucose concentrations in HFD-fed WT and MTKO mice during the fasting glucose turnover study. n = 13 WT and 7 MTKO mice. mean ±s.e.m. is plotted.
**h.-k.** The indicated parameters were measured in HFD-fed WT and MTKO mice during the fasting glucose turnover study. n = 13 WT and 7 MTKO mice. NEFA, nonesterified fatty acid
All data are presented as mean ± s.e.m. of biologically independent samples, analyzed using a two-tailed t test. NS, not significant.

bioRxiv preprint doi: https://doi.org/10.1101/2025.06.29.662135; this version posted July 3, 2025. The copyright holder for this preprint (which was not certified by peer review) is the author/funder, who has granted bioRxiv a license to display the preprint in perpetuity. It is made available under aCC-BY 4.0 International license.

# Suppl. Fig. 1

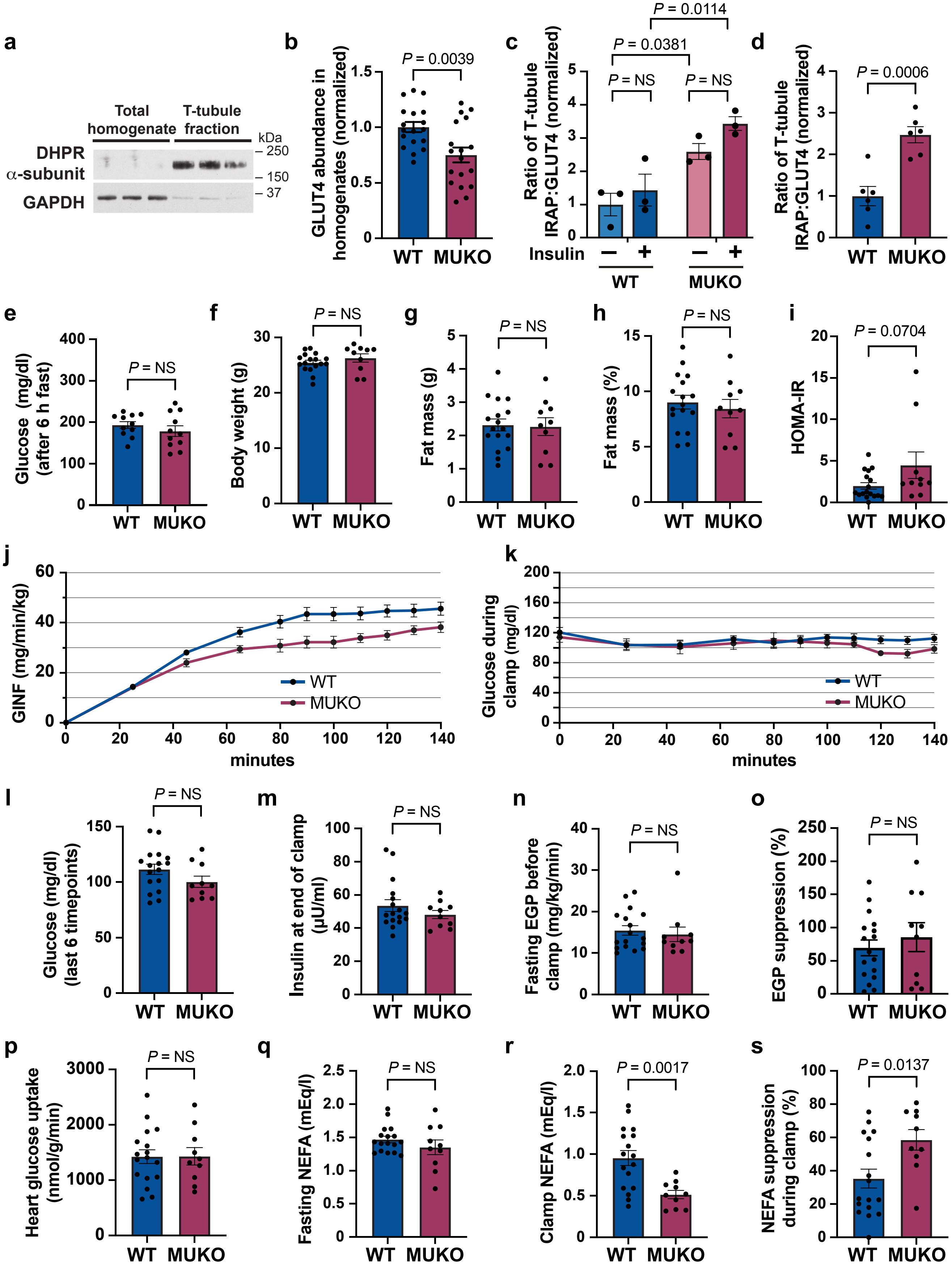

bioRxiv preprint doi: https://doi.org/10.1101/2025.06.29.662135; this version posted July 3, 2025. The copyright holder for this preprint (which was not certified by peer review) is the author/funder, who has granted bioRxiv a license to display the preprint in perpetuity. It is made available under aCC-BY 4.0 International license.

# Suppl. Fig. 2

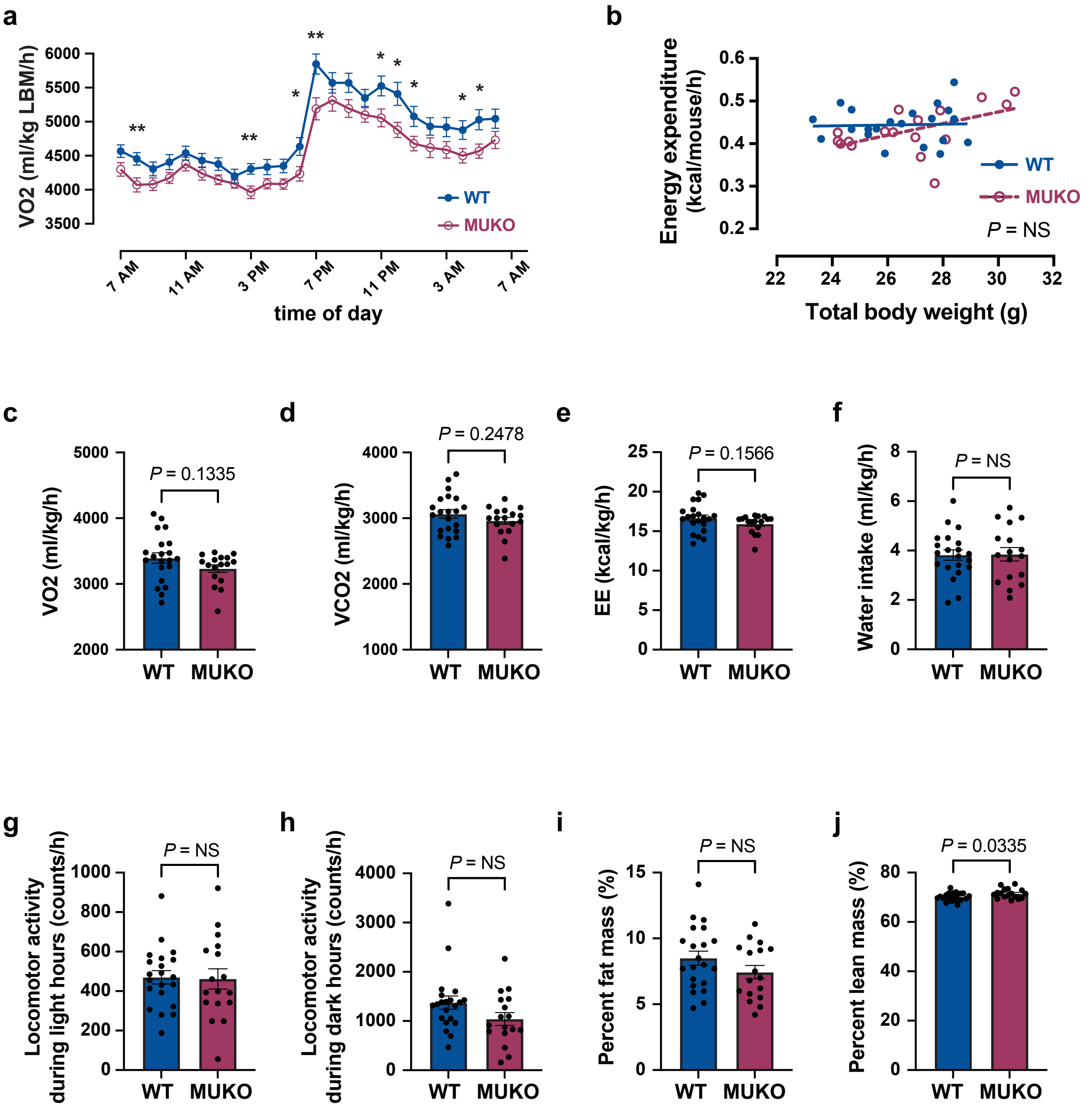

bioRxiv preprint doi: https://doi.org/10.1101/2025.06.29.662135; this version posted July 3, 2025. The copyright holder for this preprint (which was not certified by peer review) is the author/funder, who has granted bioRxiv a license to display the preprint in perpetuity. It is made available under aCC-BY 4.0 International license.

# Suppl. Fig. 3

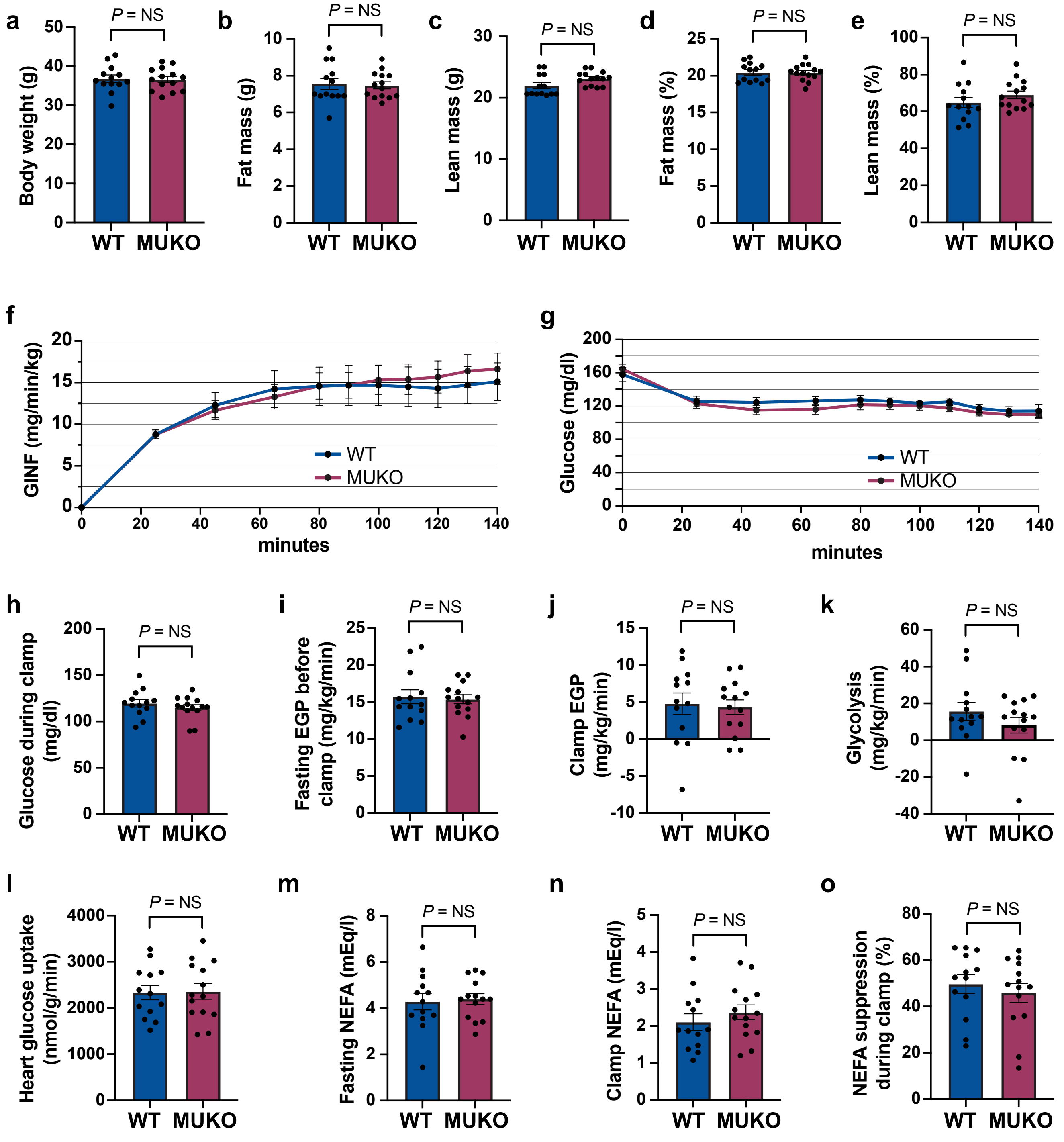

bioRxiv preprint doi: https://doi.org/10.1101/2025.06.29.662135; this version posted July 3, 2025. The copyright holder for this preprint (which was not certified by peer review) is the author/funder, who has granted bioRxiv a license to display the preprint in perpetuity. It is made available under aCC-BY 4.0 International license.

# Suppl. Fig. 4

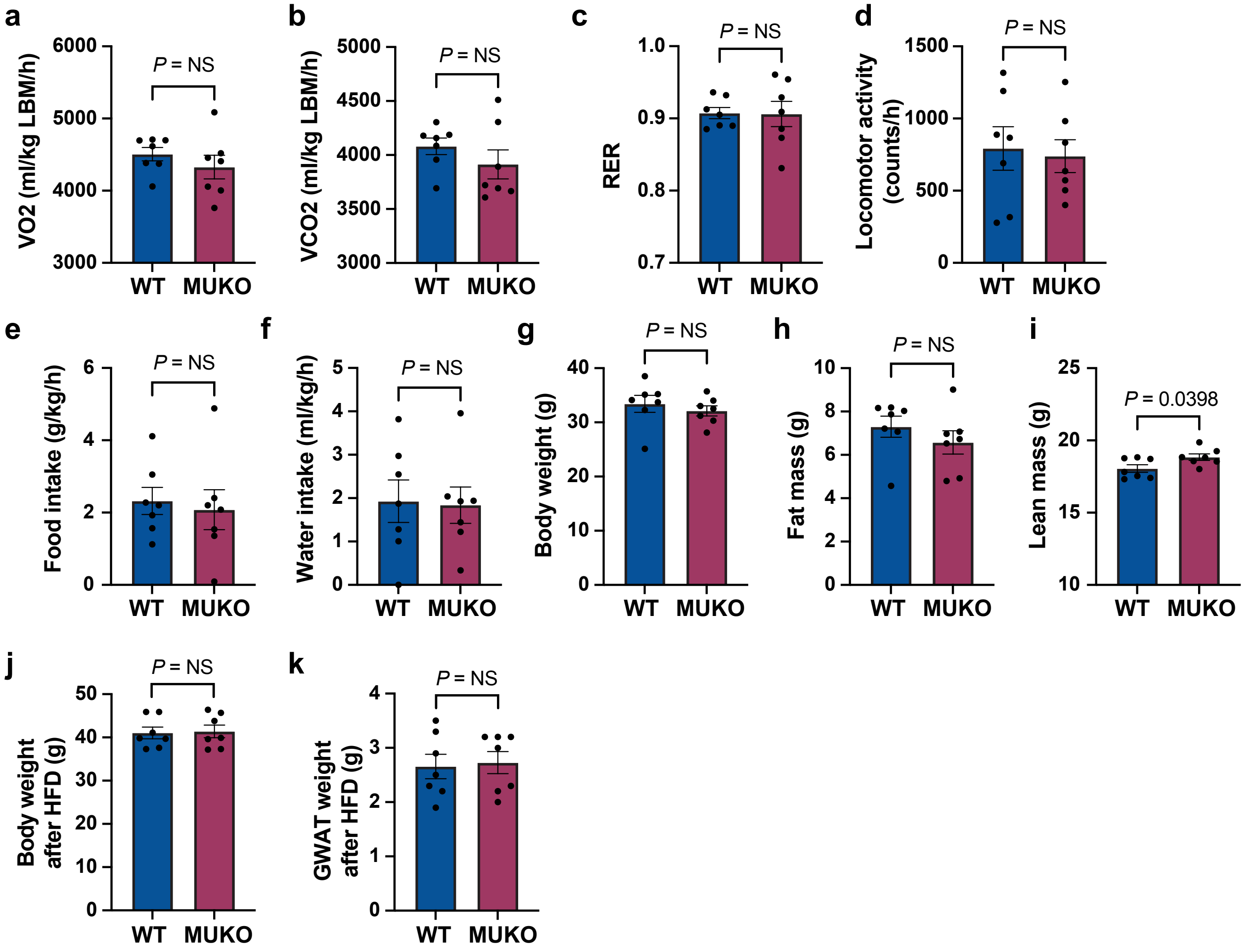

bioRxiv preprint doi: https://doi.org/10.1101/2025.06.29.662135; this version posted July 3, 2025. The copyright holder for this preprint (which was not certified by peer review) is the author/funder, who has granted bioRxiv a license to display the preprint in perpetuity. It is made available under aCC-BY 4.0 International license.

# Suppl. Fig. 5

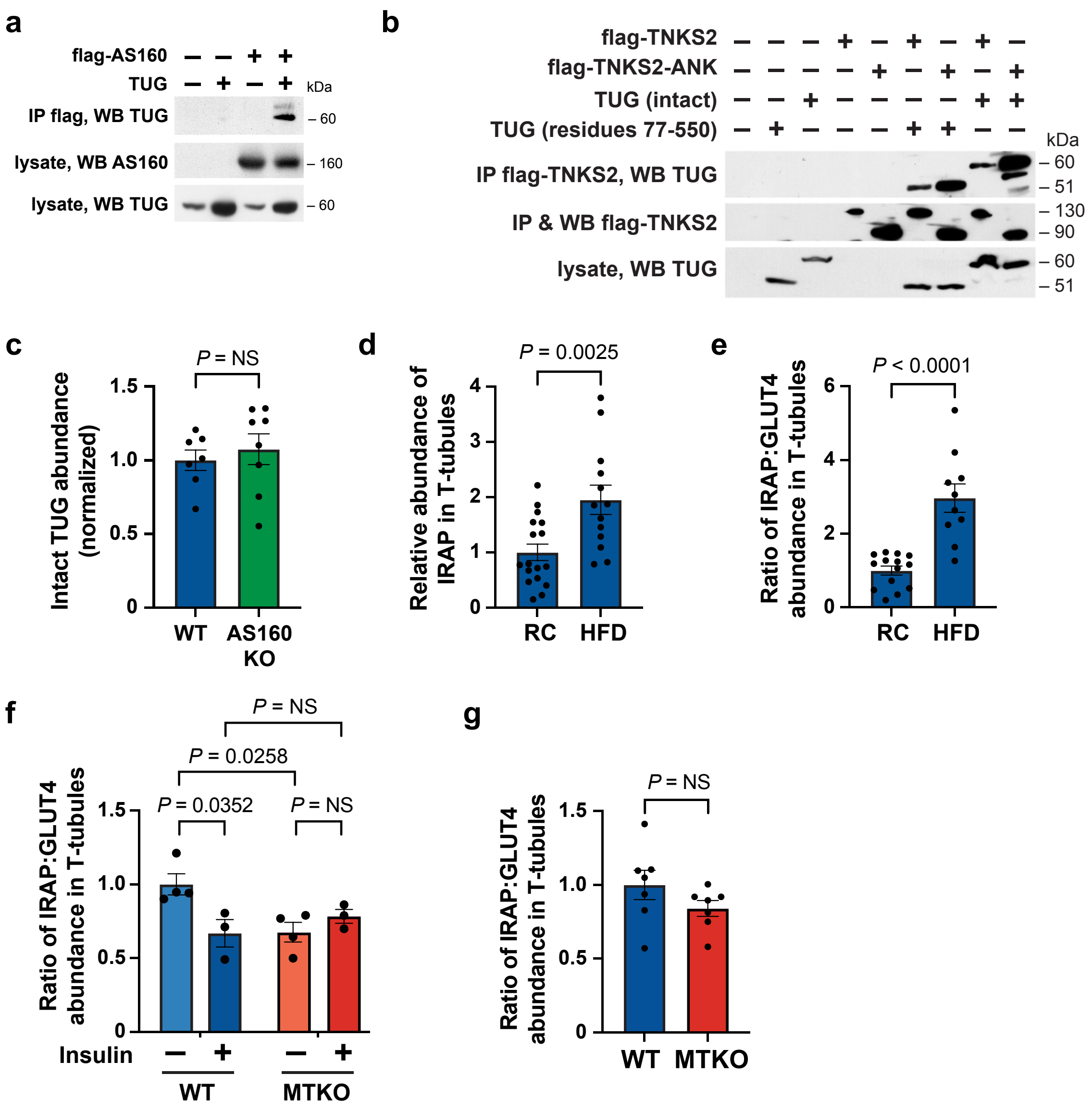

bioRxiv preprint doi: https://doi.org/10.1101/2025.06.29.662135; this version posted July 3, 2025. The copyright holder for this preprint (which was not certified by peer review) is the author/funder, who has granted bioRxiv a license to display the preprint in perpetuity. It is made available under aCC-BY 4.0 International license.

# Suppl. Fig. 6

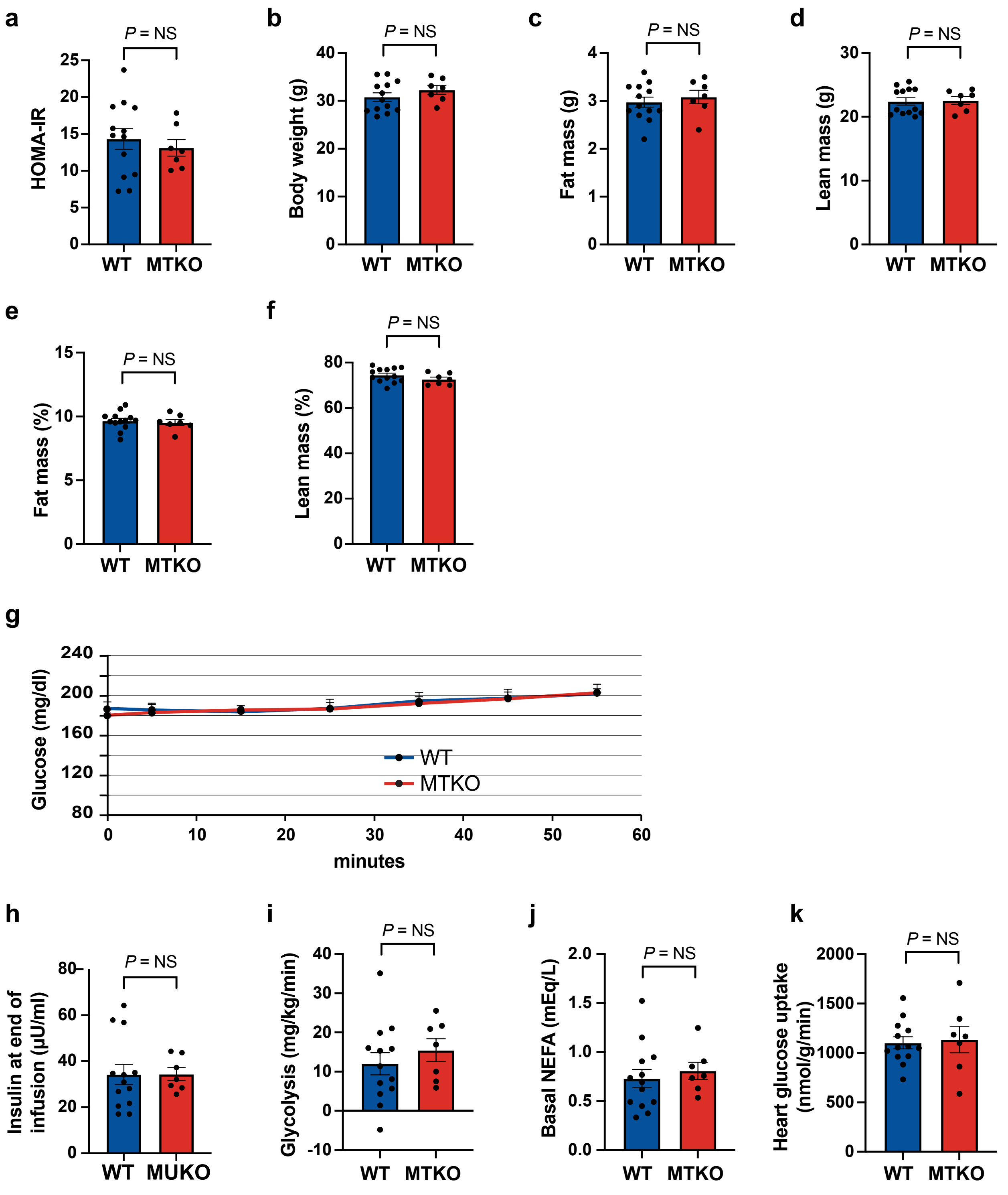

Supplement: Supplement 1 [file NIHPP2025.06.29.662135v1-supplement-1.pdf]
